# Supplementary material for: A physical memristor based Muthuswamy–Chua–Ginoux system
Source: Sci Rep. 2020 Nov 5;10:19206. doi: 10.1038/s41598-020-76108-z (PMC7645598; doi:10.1038/s41598-020-76108-z)
Supplement: Supplementary file 1 — Supplementary information. [file 41598_2020_76108_MOESM1_ESM.pdf]

A Physical Memristor Based Muthuswamy-Chua-Ginoux System

Jean-Marc Ginoux[1,*], Bharathwaj Muthuswamy[2], Riccardo Meucci[3,4], Stefano Euzzor[3], Angelo Di Garbo[5], and Kaliyaperumal Ganesan[6]

[1]Center for Theoretical Physics, CNRS, UMR 7332, Université de Toulon, France, ginoux@univ-tln.fr
[2]Quantum and Nonlinear Engineering Systems, Plainsboro, New Jersey, USA, bharath@qnes.org
[3]Istituto Nazionale di Ottica, Consiglio Nazionale delle Ricerche, Firenze, Italy, stefano.euzzor@ino.it
[4]Department of Physics and Astronomy, Università di Firenze, Firenze, Italy, riccardo.meucci@ino.it
[5]Istituto di Biofisica, Consiglio Nazionale delle Ricerche, Pisa, Italy, angelo.digarbo@pi.ibf.cnr.it
[6]School of Information Technology and Engineering, Vellore Institute of Technology Vellore 632014, Tamilnadu, India, kganesan@vit.ac.in
[*]corresponding author

ABSTRACT

Additional information

To include, in this order: **Accession codes** (where applicable); **Competing interests** (mandatory statement).

The corresponding author is responsible for submitting a competing interests statement on behalf of all authors of the paper. This statement must be included in the submitted article file.

Appendix

By considering that the *thermistor characteristics* $R(z)$ can be re-written as follows:

$$\mu z^2 + \gamma z + \theta = \mu z'^2 + \Delta \quad \text{where} \quad z' = z + \frac{\gamma}{2\mu} \quad \text{where} \quad \Delta = \frac{4\mu\theta - \gamma^2}{4\mu},$$

and with the following changes of variables and parameters:

$$x \to Ax, \quad y \to By, \quad z' \to Cz', \quad t \to Kt$$

where

$$A = \frac{1}{\sqrt{b}}\left(\frac{\eta}{\alpha}\right)^{\frac{3}{4}}, \quad B = \frac{1}{\sqrt{b}}\left(\frac{\eta}{\alpha}\right)^{\frac{1}{4}}, \quad C = \frac{1}{\sqrt{\mu}}\left(\frac{\eta}{\alpha}\right)^{\frac{1}{4}}, \quad K = \sqrt{\alpha\eta}$$

and

$$a_0 = \sqrt{\frac{\alpha}{\eta}}(a+\Delta), \quad a_1 = \frac{\sqrt{\mu}}{b}\eta\left(\frac{\eta}{\alpha}\right)^{\frac{1}{4}}, \quad a_2 = \frac{\sqrt{\mu}}{b}\Delta\sqrt{\alpha\eta}\left(\frac{\eta}{\alpha}\right)^{\frac{1}{4}}, \quad a_3 = \varepsilon\sqrt{\alpha\eta}, \quad a_4 = \frac{\gamma}{2\sqrt{\mu}}\left(\frac{\alpha}{\eta}\right)^{\frac{1}{4}}.$$

the MCG system (6) is recast in the following dimensionless form:

$$\frac{dx}{dt} = y,$$
$$\frac{dy}{dt} = -\left(x + a_0 y + y^3 + yz^2\right), \tag{1}$$
$$\frac{dz}{dt} = a_1 y^2 z^2 + a_2 y^2 - a_3 (z - a_4),$$

Let us notice that the changes of variables and parameters leads to pose $K = \sqrt{\alpha\eta}$ which is nothing else but the so-called Thomson's formula $K = \sqrt{LC}$ because we have previously posed $\alpha = C$ and $\eta = L$. This highlights the fact that this physical memristor is based on the same features as the classical ones[1,2]. By using the parameters of the NTC thermistor B57236S0250M000 ($R_0 = 60\Omega$ and $\beta = 3000K$) we deduce from

$$\alpha = C \quad ; \quad \eta = L \quad ; \quad \theta = \frac{R_0}{c} \quad ; \quad \gamma = -\frac{R_0}{c}\frac{\beta}{T_0^2} \quad ; \quad \mu = \frac{R_0}{c}\frac{\beta(\beta + 2T_0)}{2T_0^4} \quad ; \quad \varepsilon = \frac{\delta}{c}.$$

that $\mu = 1/25$, $\gamma = -2$ and $\theta = 60$. Thus, for $a = -70$, $b = 1$, $\eta = 1$, $\varepsilon = 0.6$ and $\alpha = 0.2$, i.e., $a_0 = -15.65$, $a_1 = 0.29$, $a_2 = 4.68$, $a_3 = 0.26$ and $a_4 = -3.34$, we obtain exactly the same *double spiral attractor* of period three as the one observed in Fig. 7(a) and 8(a).

Moreover, such a dimensionless system (1) can be implemented by analog simulations using the following scheme (see Fig. 1). The advantages in using analog simulations are related to the fact we can choose the appropriate temporal scale by selecting the time constant RC of the three integrators I_1, I_2 and I_3. The parameter values a_0, a_1, a_2, a_3 can be set by selecting the other resistor values according to the following conditions: $R_0 = R/a_0$, $R_1 = R/a_1$, $R_2 = R/a_2$, $R_3 = R/a_3$ and the bias voltage V according to $V = 1/(a_3 a_4)$.

References

1. Ginoux, J. M. & Rossetto, B. The singing arc: The oldest memristor? In Adamatzky, A. & Chen, G. (eds.) *Chaos, CNN, Memristors and Beyond*, 494–507 (World Scientific, Singapore, 2013).

2. Ginoux, J. M. *History of Nonlinear Oscillations Theory in France (1880-1940)* (Springer International Publishing, 2017).

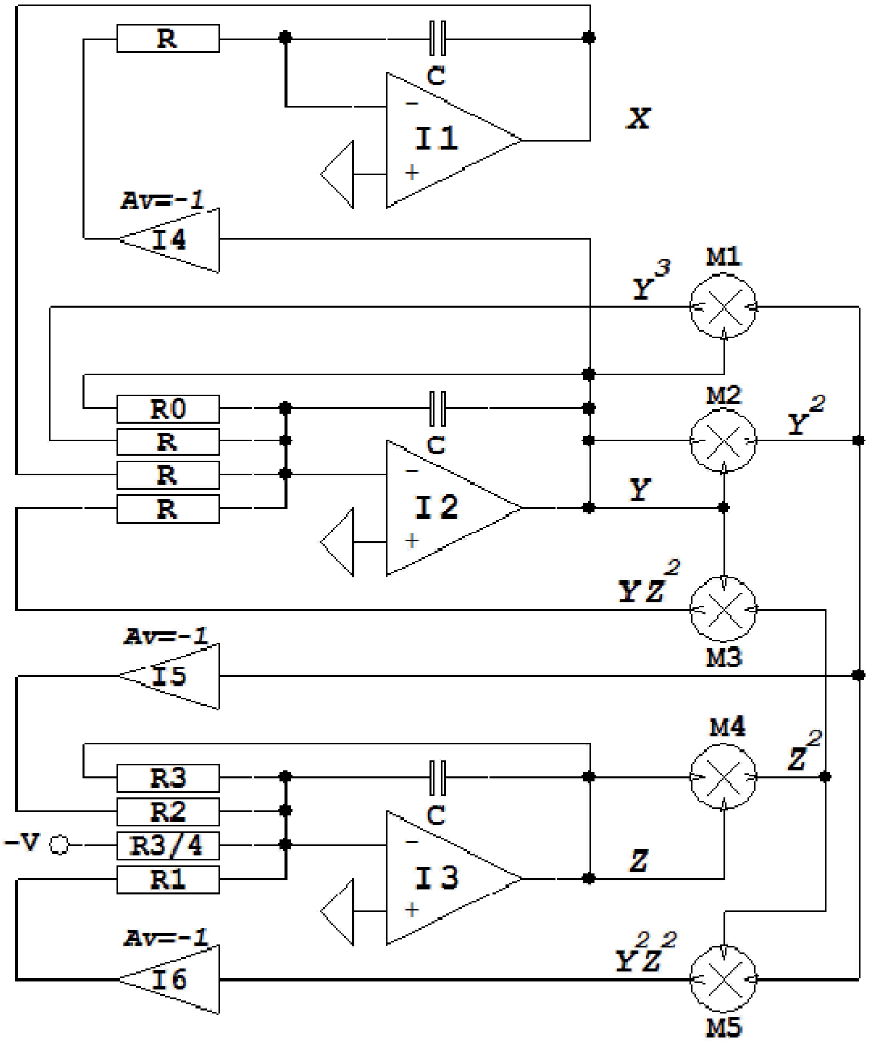

Figure 1. Schematic of the dimensionless system (1), M_1, M_2, M_3, M_4 and M_5 are analog multipliers, I_1, I_2, I_3, I_4, I_5 and I_6 are operational amplifiers.
